# Efficacy and safety of aripiprazole in child and adolescent patients

**DOI:** 10.1007/s00787-012-0270-0

**Published:** 2012-03-25

**Authors:** Eiji Kirino

**Affiliations:** 1 Department of Psychiatry, School of Medicine, Juntendo University, Tokyo, Japan; 2 Department of Psychiatry, Juntendo University Shizuoka Hospital, 1129 Nagaoka, Izunokunishi, Shizuoka 4102211 Japan; 3 Juntendo Institute of Mental Health, Saitama, Japan

**Keywords:** Aripiprazole, Child, Adolescent, ADHD, Pervasive development disorder, Asperger’s syndrome

## Abstract

Aripiprazole (APZ) has a unique pharmacological profile, as a partial agonist at the dopamine D2 and serotonin 5HT1A receptors and an antagonist at the serotonin 5HT2A receptor; this drug has few side effects (such as extrapyramidal syndrome, hyperprolactinemia, weight gain, metabolic disorders, and sedation) which are typical problems with other antipsychotic drugs. Due to its high tolerability, it is possible to safely administer it to children and adolescents. Efficacy and tolerability of APZ in children and adolescents have been well demonstrated in many clinical studies, which supported approvals granted by the US Food and Drug Administration (FDA) for schizophrenia, bipolar diseases, and irritability associated with autistic disorder in children and adolescents. APZ is expected to exert sedative, anti-depressive, and anti-anxiety effects, and stabilize emotion. APZ is an antipsychotic drug which could be useful for a wider spectrum of psychiatric disorders in children and adolescents. There is little risk of deterioration (such as disinhibition and acting out) and rapid stabilization is easy to achieve in children and adolescents without definitive diagnoses or with a combination of more than one spectrum of disorders. The effectiveness of APZ in children and adolescents is reviewed and discussed, given its pharmacological profile and the outcomes of various clinical studies. However, randomized or blind studies are still limited, and the majority of reports referenced here are open-label studies and case reports. Conclusions drawn from such studies must be evaluated with caution, and a further accumulation of controlled studies is thus needed.

## Introduction

Aripiprazole (APZ) is an antipsychotic drug approved by the US Food and Drug Administration (FDA) for schizophrenia in 2002. Since then, it has been approved worldwide including Japan in 2006 for treatment of schizophrenia. The efficacy and tolerability of APZ, a dopamine D2 partial agonist [1] distinctive from conventional antipsychotics, have been demonstrated in various psychotic diseases. APZ is indicated in the USA for treatment of schizophrenia and bipolar I disorder, and has been recently approved for the treatment of irritability associated with autistic disorder in children and adolescents. The effectiveness of APZ in children and adolescents is reviewed and discussed herein, given its pharmacological profile and the outcomes of various clinical studies.

## Pharmacological profile

### Affinity for dopamine D2 receptors and intrinsic activity

APZ is classified as a third-generation atypical antipsychotic drug because of its unique mechanism of action distinctive from that of other atypical antipsychotics [2]. APZ functions as a dopamine D2 partial agonist and appropriates intrinsic activity at dopamine D2 receptors, stabilizing dopamine D2 receptor-mediated neurotransmission without excessive blocking. That is why APZ is often called a dopamine system stabilizer [3]. APZ, which has higher affinity at dopamine D2 receptors than endogenous dopamine, works as a partial agonist. Therefore, APZ is less likely to induce extrapyramidal symptoms (EPS) and elevate serum prolactin levels compared to other antipsychotics. There are a few other partial agonists that have been demonstrated to be effective in reducing negative symptoms and lowering the incidence of EPS, but none of them has exhibited non-inferiority over typical antipsychotics in other psychotic symptoms such as positive symptoms [4–9]. Differences between APZ and other partial agonists can be ascribed to the extent of intrinsic activity at dopamine D2 receptors. APZ is also a high-affinity partial agonist at dopamine D3 receptors [4, 10].

### Affinity to serotonin 5HT receptors

APZ has high affinity to serotonin 5HT1A and 5HT2A receptors. 5HT1A knockout mice exhibit anxiety behavior [11], and 5HT1A receptor agonists appear to have anti-anxiety and anti-depressive effects and improve cognitive function [12]. APZ is a partial agonist of serotonin 5HT1A, which is expected to improve anxiety and depression and to reduce antipsychotic-derived EPS [13, 14]. de Quervain et al. [15] found that genetic variations of 5HT2A receptors affect human episode memory, which indicates that the 5HT2A receptor is closely related with cognitive function [16]. APZ is a high-affinity antagonist at serotonin 5HT2A receptors, decreasing 5HT2A receptor activity, which may improve negative symptoms and reduce antipsychotics-induced EPS [17].

Wirshing et al. [18] indicated that the second-generation antipsychotics [such as clozapine, risperidone (RIS) and olanzapine (OLZ)] which block serotonin 5HT receptors and histamine receptors are associated with body weight gain. Sargent et al. [19] reported that *m*-chlorophenyl piperazine (*m*-CPP), a 5HT2C receptor agonist, may continue to activate brain 5-HT2C receptors and this effect is associated with decreases in appetite and body weight. Nasrallah [20] suggested that generic variations of 5HT2C might affect antipsychotics-derived weight gain, because OLZ and RIS significantly increased weight in drug-naïve patients with 5HT2C polymorphism compared with wild-type patients. Therefore, 5HT2C antagonists are associated with weight gain, while APZ, a 5HT2C receptor partial antagonist, appears to regulate appetite with less effect on weight and an anti-obesity effect.

### Affinities for other receptors

Drugs that are high-affinity antagonists at histamine H1 receptors are associated with weight gain or hypersedation [21–23], while high-affinity antagonists at muscaline M1 receptors are related to anticholinergic side effects such as dry mouth, constipation, and blurred vision [23–25]. APZ is an antagonist, but has a low affinity at histamine H1, adrenaline α1 [21], and muscaline M1 receptors [22] and few side effects [26], as described in the guidelines of American Psychiatric Association (APA) [27].

## Efficacy in children and adolescents

APZ is indicated in the USA for acute and maintenance treatment of schizophrenia in adolescents (13–17 years of age) and acute treatment of bipolar I disorder in children (10–17 years of age) as monotherapy and as an adjunctive to lithium or valproate. It is also indicated for use in treating irritability associated with autistic disorder in pediatric patients (6–17 years of age) [28–32]. Aparasu et al. [33] examined patterns and determinants of antipsychotic prescribing in children and adolescents receiving outpatient care in the USA. They analyzed outpatient visits involving 11 typical and 6 atypical antipsychotic agents prescribed for children and adolescents. Most (99 %) of these visits involved prescribing of atypical agents, and the most frequently prescribed atypical agents were RIS, QTP, and APZ.

### Schizophrenia

Findling et al. [34] reported the results of a 6-week, multicenter, double-blind, randomized, placebo-controlled trial in which children and adolescents (13–17 years of age, *n* = 302) with schizophrenia and a PANSS total score of 70 or more were randomly assigned (1:1:1 ratio) to placebo, 10 mg/day of APZ, or 30 mg/day of APZ. At the end of the study, both APZ doses showed statistically significant differences from placebo in reduction in PANSS total score and were well tolerated in all patients.

Kirino [35] reported that APZ was effective in a 15-year-old schizophrenic patient with long-term catatonic stupor. This patient was first given RIS 0.5–2 mg/day and switched to APZ 6 mg/day due to hypersedation. After the APZ dose was increased to 12 mg/day, the patient started to speak more; he had more active communications after the dose was increased to 18 mg/day without any adverse events. This case suggests a unique effect of APZ as a dopamine D2 receptor partial agonist distinctive from other antipsychotics, with reduced risk of hypersedation, and as a serotonin 5HT1A receptor partial agonist that is useful in treating catatonic anxiety. APZ may be effective in the treatment of some schizophrenic patients with stupor, which sometimes carries a risk of physical debilitation and requires special attention due to the risk of adverse drug reactions.

### Bipolar I disorder

Tramontina et al. [36] assessed the response to treatment with APZ in children and adolescents with bipolar disorder comorbid with attention-deficit/hyperactivity disorder (ADHD) in a 6-week double-blind, placebo-controlled trial. The group receiving APZ (8–17 years of age, *n* = 18) showed a significantly greater reduction in the Young Mania Rating Scale (YMRS) scores, the Child Mania Rating Scale-Parental Version (CMRS-P) scores, and the Clinical Global Impressions-Severity (CGI-S) scores from baseline to end point when compared with the placebo group (*n* = 25). In addition, higher rates of response and remission were found for the APZ group. No significant between-group differences were found in weight, ADHD symptoms, or depressive symptoms. Adverse events significantly more frequent in the APZ group were somnolence and sialorrhea. APZ was effective in reducing manic symptoms and improving global functioning without promoting severe adverse events or weight gain. However, conclusions drawn from such a study including subjects with comorbid condition must be evaluated with caution.

Findling et al. [30] carried out a randomized, double-blind, placebo-controlled trial with a follow-up period of 4 weeks in 296 cases aged 10–17 years with bipolar disorder type I (mania) and with a mixed episode. They reported that APZ 10 mg/day or 30 mg/day significantly improved the YMRS total score by 1 week and the response rate (a reduction in the YMRS total score by 50 % or more) 4 weeks later was significantly higher than in the placebo cases; this suggested that APZ was effective for bipolar disorder at the acute stage in childhood and adolescence.

### Pervasive development disorder (PDD)

In an 8-week double-blind, randomized, placebo-controlled, parallel-group study reported by Marcus et al., 218 children and adolescents (6–17 years of age) with autistic disorder and behaviors such as tantrums, aggression, self-injurious behavior, or a combination of these symptoms, were randomized 1:1:1:1 to APZ (5, 10, or 15 mg/day) or placebo. All APZ doses demonstrated significantly greater improvements than placebo in mean Aberrant Behavior Checklist Irritability (ABC-I) subscale scores and mean CGI-I score than placebo at week 8. The most common adverse event leading to discontinuation was sedation. There were two serious adverse events: presyncope (5 mg/day) and aggression (10 mg/day). At week 8, mean weight change was as follows: placebo +0.3 kg, APZ 5 mg/day +1.3 kg, APZ 10 mg/day +1.3 kg, and APZ 15 mg/day +1.5 kg; all *p* < 0.05 versus placebo. APZ was efficacious and generally safe and well tolerated in the treatment of children and adolescents with irritability associated with autistic disorder [37].

In a 14-week, prospective, open-label study [38], APZ was administered to 25 children and adolescents (5–17 years of age) diagnosed with pervasive developmental disorder not otherwise specified (PDD-NOS) or Asperger’s syndrome (range: 2.5–15.0 mg/day, mean dose: 7.8 mg/day). Full-scale intelligence quotient (IQ) scores ranged from 48 to 122 (mean 84). Twenty-two of 25 subjects (88 %) were responders with regard to interfering symptoms of irritability, including aggression, self-injury, and tantrums, with a final CGI-I of 1 or 2 (very much or much improved) and a 25 % or greater improvement on the Aberrant Behavior Checklist (ABC-I). The final mean CGI-I was 1.6. ABC-I scores ranged from 18 to 43 (mean 29) at baseline, whereas scores at week 14 ranged from 0 to 27 (mean 8.1). APZ was well tolerated. Mild EPSs were reported in nine subjects (36 %). Body mass index (BMI) increased from 20.3 at baseline to 21.1 at end point. Prolactin significantly decreased from 9.3 to 2.9 ng/mL. These data suggest that APZ may be effective and well tolerated for severe irritability in pediatric patients with PDD-NOS or Asperger’s syndrome. In a retrospective naturalistic study in 34 patients (4.5–15 years of age) with severely impaired PDD [39], the mean baseline CGI-S was 5.7 (markedly ill/severely ill). The mean final dosage of APZ was 8.1 mg/day. At the end point, 11 patients (32.4 %) were ‘much improved’ or ‘very much improved’ (CGI-I score of 1 or 2), 12 patients (35.3 %) were ‘minimally improved’ (CGI-I score of 3), and 10 (29.4 %) were ‘unchanged’ or ‘worsened’ (CGI-I score of 4 or 5). The Children’s Global Assessment Scale (C-GAS) and the Childhood Autism Rating Scale (CARS) scores significantly improved. Nine patients (26.5 %) experienced moderate to severe agitation, which was associated with self-injurious behaviors in five of these patients, and five patients (14.7 %) presented with sleep disorders. Twelve patients (35.3 %) discontinued medication during the follow-up because of lack of efficacy or adverse effects. In these severely impaired children with PDDs, APZ monotherapy was associated with a significant improvement in maladaptive behaviors in one-third of patients. Agitation and insomnia were the most frequent adverse effects.

### Attention-deficit hyperactivity disorder (ADHD)

In a 6-week open-label study in ADHD patients (combined type and predominately inattentive type) (8–12 years of age, *N* = 23) [40], end of study results showed overall significant improvement from baseline on ADHD and functional outcome measures at a mean dose of 6.7 mg/day. No significant differences from baseline performance were found on the cognitive measures at the end of the study. The most frequently reported adverse events were sedation (78.3 %) and headache (47.8 %). In this cohort, APZ led to clinical benefit in reducing ADHD symptoms and improving overall functioning. Of note, cognitive functioning did not appear to be negatively impacted by APZ treatment.

### Tic disorders

Yoo et al. [41] investigated the efficacy and tolerability of APZ for treating children and adolescents with tic disorders in an 8-week open-label study using a flexible dosing schedule (range: 2.5–20 mg/day, mean dose: 9.8 mg/day) in 24 outpatients (7–18 years of age). There was a 52.5 % reduction in the mean score of the Korean versions of the Yale Global Tic Severity Scale (YGTSS) Total Tic scores. Nineteen patients (79.2 %) showed either much improved or very much improved status according to the CGI-I. The CGI-S score was also reduced (from 5.5 to 3.0). The initial dose of 5 mg/day APZ for 2 weeks was also found to reduce tic symptoms significantly. This study suggests that APZ is an efficacious and safe treatment for children and adolescents with tic disorders. Seo et al. [42] conducted a study to evaluate the effectiveness of APZ to reduce the severity and frequency of tic symptoms and to evaluate the additional effects of APZ on weight changes in children and adolescents with Tourette’s syndrome or chronic tic disorders. A 12-week, open-label trial with flexible dosing strategy of APZ was performed with 15 participants (7–19 years of age). Significant decreases in the scores of motor and phonic tics, global impairment, and global severity were demonstrated between baseline and week 3, and the scores continued to improve thereafter. No difference was observed between the baseline and end point body mass index (BMI) values. This study demonstrates that a relatively low dose of APZ can be used to control tic symptoms effectively in children and adolescents with Tourette’s syndrome and chronic tic disorders without causing significant weight gain.

### Delusional disorders

Myers et al. [43] treated a 17-year-old female diagnosed with delusional disorder (erotomanic type with a history of seven episodes of stalking) with APZ 10 mg/day combined with cognitive behavior therapy and family therapy. As a result of treatment, she became better able to communicate with others, with continued improvement at month 5, and was able to avoid a contact with the stalking victim. She is still being treated with APZ 10 mg/day; the drug has been well tolerated. They concluded that combination treatment with APZ and psychological therapy are effective in addressing delusional disorders.

### Obsessive–compulsive disorder (OCD)

Storch et al. [44] reported a 13-year-old male with OCD who had an incomplete response to combined cognitive-behavioral therapy (CBT) and sertraline before successful augmentation of CBT with APZ 2.5–5 mg/day. Standardized assessments indicated significant reductions in OCD symptomatology associated with both initial treatment and APZ augmentation. This case suggests that APZ may have utility as an augmenting agent of CBT in adolescents with OCD by virtue of its dual impact on serotonergic and dopaminergic mechanisms.

Reported clinical and safety outcomes of APZ in children and adolescents referenced above are summarized in Table 1.

**Table 1:** Summary of reported clinical and safety outcomes of APZ in children and adolescents

Study	Age (years)	Study design	Subjects (*N*)	Clinical outcomes	Safety outcomes	Dosage of APZ and other therapy
Findling et al. [34]	13–17	A 6-week multicenter, double-blind, randomized, placebo-controlled trial	Schizophrenia (302)	APZ showed significant differences from placebo in reduction in PANSS total score	Well tolerated in all patients	10 or 30 mg/day
Tramontina et al. [36]	8–17	A 6-week double-blind, randomized, placebo-controlled trial	Bipolar disorder comorbid with ADHD (18)	APZ was effective in reducing manic symptoms and improving global functioning	No severe adverse events or weight gain	Initial: 2 (BW < 50 kg) or 5 (BW > 50 kg) Maximum: 20 mg/day Mean: 13.61 mg/day
Findling et al. [30]	10–17	A 4-week randomized, double-blind, placebo-controlled trial	Bipolar I (mania) and a mixed episode (296)	APZ significantly improved the YMRS total score and the response rate was significantly higher than in the placebo cases	Generally well tolerated	10 or 30 mg/day
Marcus et al. [37]	6–17	An 8-week randomized, placebo-controlled, parallel-group study	Autistic disorder and behaviors such as tantrums, aggression, self-injurious behavior, or a combination of these symptoms (218)	APZ demonstrated significantly greater improvements than placebo in ABC-I subscale scores and CGI-I score than placebo	Adverse event leading to discontinuation: sedation. Serious adverse events: presyncope (0.46 %) and aggression (0.46 %)	5, 10, or 15 mg/day
Stigler et al. [38]	5–17	A 14-week prospective, open-label study	PDD-NOS or Asperger’s syndrome (25)	APZ demonstrated improvements on the CGI-I and ABC-I. APZ	APZ was well tolerated. Adverse event: mild EPSs (36 %)	2.5–15.0 mg/day, mean: 7.8 mg/day
Masi et al. [39]	4.5–15	A retrospective naturalistic study	Severely impaired PDD (34)	APZ demonstrated improvements on the CGI-I, C-GAS and CARS	Adverse events: moderate or severe agitation (26.5 %) and sleep disorders (14.7 %)	Mean: 8.1 mg/day
Findling et al. [34]	8–12	A 6-week open-label study	ADHD (combined type and predominately inattentive type) (23)	APZ demonstrated overall significant improvement on ADHD and functional outcome measures	Cognitive functioning did not appear to be negatively impacted by APZ Adverse events: sedation (78.3 %) and headache (47.8 %)	Mean: 6.7 mg/day
Yoo et al. [41]	7–18	An 8-week open-label study with flexible dosing schedule	Tic disorder (24)	APZ demonstrated improvements on the CGI-I, CGI-S, and YGTSS	Adverse events: hypersomnia (37.5 %), nausea (20.8 %), and headache (16.6 %)	2.5–20 mg/day, mean: 9.8 mg/day
Seo et al. [42]	7.3–19.2	A 12-week, open-label trial with flexible dosing	Tourette’s syndrome or chronic tic disorders (15)	Significant decreases in the scores of motor and phonic tics, global impairment, and global severity were demonstrated	No difference between the baseline and end point BMI values	Initial: 2.5–7.5 mg/day Mean final: 8.17 mg/day (0.20 mg/Kg/day)

## Safety data in children and adolescents

### Body weight and metabolic parameters

Correll et al. [45] carried out a cohort study to evaluate cardiometabolic effects of the second-generation antipsychotics in children and adolescents (4–19 years of age) naive to antipsychotic medication. After a median of 10.8 weeks of treatment, mean body weight increased by 8.5 kg with OLZ (*n* = 45), 6.1 kg with QTP (*n* = 36), 5.3 kg with RIS (*n* = 135), and 4.4 kg with APZ (*n* = 41) compared with the minimal weight change of 0.2 kg in the untreated comparison group (*n* = 15). With OLZ and QTP, respectively, the mean levels increased significantly for total cholesterol (15.6–24.3 and 9.1–17.7 mg/dL), triglycerides (24.3–38.9 and 37.0–63.8 mg/dL), non-HDL cholesterol [total cholesterol minus HDL cholesterol] (16.8–24.3 and 9.9–18.4 mg/dL), and the ratio of triglycerides to HDL cholesterol (0.6–0.9 and 1.2–2.0). With RIS, triglycerides increased significantly (9.7–19.0 mg/dL). Metabolic baseline-to-endpoint changes were not significant with APZ or in the untreated comparison group.

### EPS and akathisia

In randomized, double-blinded, placebo-controlled trials of schizophrenia [34], bipolar type I disorder [30], and autism [31, 37] in childhood and adolescence, it was reported that the occurrence of EPS was more frequent in the APZ group than in the placebo group and salivation, tremor, and dystonia were observed. However, the degree of EPS was considered mild to moderate.

Akathisia was more frequently observed in the high-dose APZ group (30 mg/day), but it was comparably observed between the group receiving APZ at 10 mg/day and the placebo group [34]. Findling et al. [30] reported that the occurrence rate of akathisia was higher in the group of patients receiving APZ 10 mg/day or greater when compared with the control group. Moreover, there was another report that the occurrence rate was comparable between the APZ and placebo groups [31, 37]. Therefore, no consensus has been reached as to the occurrence rate of akathisia in patients taking APZ.

However, since the approved dose of APZ is 10 or 30 mg/day for schizophrenia and bipolar disorder cases and 5, 10, or 15 mg/day for autism cases, there is a possibility that the occurrence rate of akathisia is higher in cases receiving a higher dose of APZ.

### Sedation

Among the randomized, double-blinded, placebo-controlled trials, sedation was more frequently observed in the APZ group than in the placebo group in trials on autism [31, 37]. No adverse event of sedation was reported in cases with schizophrenia [34] and bipolar disorder [30], but the sleepiness appeared more frequently in the APZ group than in the placebo group. It was considered that a higher dose of APZ induced a higher rate of sedation and sleepiness in cases with these disorders.

### Serum prolactin

The serum prolactin level was significantly lower after APZ administration than at baseline, and it was also significantly lower in the APZ group than in the placebo group in the randomized, double-blinded, placebo-controlled trials for schizophrenia [34], bipolar type I disorder [30], and autism [31, 37].

### Other parameters

There were no changes evident in blood pressure, heart rates, ECG, or the QTc interval in any of the double-blinded, placebo-controlled trials [31, 34, 37].

## Conclusion

APZ is expected to exert sedative, anti-depressive, and anti-anxiety effects, and to stabilize emotion. Due to its high tolerability, it is possible to safely administer it to children and adolescents. There is little risk of deterioration (such as disinhibition and acting out) and rapid stabilization is easy to achieve in children and adolescents without definitive diagnoses or with a combination of more than one spectrum of disorders. In particular, since the safety of antidepressants is not established in child and adolescents, the utility of the antidepressant effect of APZ is high. It is possible to make quality of life (QOL) improvement treatment targets with APZ administration, as well as positive symptoms in cases of irritability, aggression, anxiety, or depression.

The expert consensus guidelines on adherence problems in patients with mental illness published in 2009 discussed the side effects of medication as a factor in poor adherence to medication treatment; in particular, weight gain, sedation, akathisia, and sexual dysfunction were considered important factors leading to adherence problems [46]. Therefore, medication with a lower risk of these side effects appears to have a great impact on patient outcomes. The use of an antipsychotic agent with fewer side effects might be more critical in children and adolescents than adults to allow for continued treatment. APZ has few side effects (such as EPS, hyperprolactinemia, weight gain, metabolic disorders, and sedation) which are typical problems with other antipsychotic drugs. Furthermore, the ameliorative effects of APZ on cognitive function and communication ability consequently led to improvement of QOL. The efficacy and tolerability of APZ in children and adolescents have been well demonstrated in many clinical studies. APZ is an antipsychotic drug which could be useful for a wider spectrum of psychiatric disorders in children and adolescents. However, randomized or blind studies are still limited, and the majority of reports referenced here are open-label studies and case reports. Conclusions drawn from such studies must be evaluated with caution, and further accumulation of controlled studies is thus needed.
